# Mental Hygiene, or an Examination of the Intellect and Passions, Designed to Illustrate Their Influence on Health and the Duration of Life

**Published:** 1843-10

**Authors:** 


ART. VIII.—*Mental Hygiène, or an examination of the Intellect and Passions, designed to illustrate their influence on Health and the duration of Life.* By W. SWEETSER, M.D.—*New York*, 1843. 8vo, pp. 270.

THIS volume, though containing little not to be found in works of a similar kind, is yet an intelligent, entertaining, and a popular exposition of the relative influences of the intellectual and moral, and the bodily operations. The subjects are illustrated by a variety of facts in natural history, by apposite physiological references and explanations, and by a number of interesting anecdotes. The volume, in short, is one which, while it is not useless to the professional reader, will be perused with profit and pleasure by the public.

